# 

**Published:** 1914-07-04

**Authors:** 


### CONTENTS.

PAGE

**Hospital Saturday, the Truck Act and Workmen's Contributions** ... ... ... ... 365

**A Ministry of Public Health** ... .. ... 366

**Hospital and Institutional News ...** ... ... 367

Their Majesties' Visit to Glasgow—The Glasgow Royal Infirmary—Radium for Manchester—Manchester's Central Accident Hospital—Sir A. L. Jones Memorial Hospital—Personal Service in North Staffordshire—The Jurisdiction of the Charity Commissioners—Medical Representation upon the London Insurance Committee—Parliamentary Grants for Feeding School Children—Dr. Salter and the London Insurance Committee—Scarlet Fever in the Metropolis—British Hospital for Mothers—Walsall Workpeople and their Hospital—The Finances of the Macclesfield Infirmary—The Great Yarmouth General Hospital—A New Hospital Called for—New O.-P. Department at the Derbyshire Children's Hospital—The Chelsea Women's Hospital Scheme — The Voluntary Aid Detachments — Another "Royal" Cot and an "Army" Cot—The New Superintendent of Montrose Royal Asylum—The Metropolitan Hospital Saturday Fund—Hospital Collection on a Racecourse—This Week's Drug Market.

PAGE

**Aerial Conveyance of Infectious Diseases** ... 73

A Hint for Fever Hospital Administrators.

[See over.]

### CONTENTS—(*continued*).

PAGE

**The Colour Problem in Hospital Life ... ... 374**
Coloured Students : Factors in the Controversy.

**The Doctors' Wives Association ... ... ... 374**

**The British Hospitals Association ... ... 375**

**Voluntary Hospitals in Relation to Social Service Work ... ... ... ... 375**
By COUNCILLOR HERBERT SHAW, B.A., Sheriff of Newcastle-upon-Tyne.
The Discussion on Councillor Shaw's Paper.

**Pensions for Hospital Employees ... ... 377**
By P. J. MICHELLI, C.M.G., Secretary Seamen's Hospital Society, Greenwich, and the London Schools of Tropical and Clinical Medicine.
The Discussion on Mr. Michelli's Paper.

**Proposed Amendment of the Truck Acts, 1831 to 1896, and how it may Affect the Voluntary Hospitals ... ... ... 390**
By Mr. WILLIAM STRAKER, Corresponding Secretary Northumberland Miners' Association.
The Discussion on Mr. Straker's Paper.
The Business Meeting.
The Next Conference.

PAGE

**New Royal Hospital for Sick Children, Glasgow ... ... ... ... ... ... 379**
Some Details of the Scheme.
Description of the Buildings (from a Correspondent).
Ground Floor Plan.

**The Editor's Letter-Box ... ... ... ... 394**

The Bath Workhouse Infirmary—Institutional Defalcations.

**The National Medical Union Dinner ... ... 396**

**The Institutional Worker ... ... (Suppl.) 1**
An Appeal Showing Brains and Originality.
Stationery and Printing Contracts.
Questions for July.
Questions Invited.
Employment and Training.
The Sick and in Need.
Acknowledgments.

